# Why Don’t You [Government] Help Us Make Healthier Foods More Affordable Instead of Bombarding Us with Labels? Maternal Knowledge, Perceptions, and Practices after Full Implementation of the Chilean Food Labelling Law

**DOI:** 10.3390/ijerph19084547

**Published:** 2022-04-09

**Authors:** Teresa Correa, Camila Fierro, Marcela Reyes, Lindsey Smith Taillie, Francesca Renee Dillman Carpentier, Camila Corvalán

**Affiliations:** 1 School of Communication, Diego Portales University, Vergara, Santiago 240, Chile; teresa.correa@udp.cl (T.C.); camila.fierro@mail.udp.cl (C.F.); 2 Institute of Nutrition and Food Technology, University of Chile, El Líbano 5524, Chile; mreyes@inta.uchile.cl; 3 Department of Nutrition, Gillings School of Global Public Health, University of North Carolina, Chapel Hill, NC 27599-7461, USA; taillie@unc.edu; 4 Carolina Population Center, University of North Carolina, Chapel Hill, NC 27516, USA; 5 School of Media and Journalism, University of North Carolina, Chapel Hill, NC 27599, USA; francesca@unc.edu

**Keywords:** Chile Labelling law, knowledge, perception and practices of nutrition policies, focus groups

## Abstract

Experimental and real-life evaluations show that the use of front-of-package warning labels (FoP) in unhealthy foods is well understood and can modify people’s behaviors. However, it is unclear whether these effects remain in the long term because of the risk of message fatigue. The purpose of this study is to explore after four years of implementation of the Chilean Food Labelling law people’s dietary behavior and FoP labels attention. Nine focus groups of mothers (7–10 people each) of children (2–14 yo) were conducted in Santiago, Chile, and macrocodes were developed, combining an iterative process of deductive and inductive thematic analyses. We found that mothers experienced labels’ fatigue but also had greater knowledge about nutrition and appreciation for more natural foods. This greater knowledge about better nutrition interferes with the perception that healthier and less processed foods are financial and physically inaccessible. The key role of schools as an environment for promoting healthier diets in children was strengthened by the mothers. These results suggest that policies based on providing consumer information need reinforcement campaigns to maintain their effectiveness and that we also need to advance policies to improve access and affordability of healthy foods to ensure better diets.

## 1. Introduction

Food environment policies including improving consumer information, marketing restrictions, healthier school environments, and food and beverages taxes are key pillars for promoting healthier diets among the population. The Latin-American region has been particularly relevant in promoting these policies [1].

In June 2016, Chile launched one of the most comprehensive food environment regulations across the world that considers the use of a front-of-package (FoP) warning label for pointing out unhealthy foods, comprehensive marketing restrictions for unhealthy foods and prohibition of availability of these “high in” foods and beverages in schools [2]. Following Chile’s footsteps, several countries have recently implemented regulations that include at least one aspect of the Chilean policy. For instance, Peru, Uruguay, and Mexico have implemented mandatory black stop FoP labels for unhealthy foods. Peru also included marketing restrictions on regulated foods, and Jamaica regulated school food environments. At the same time, several other countries worldwide are discussing the implementation of similar policies such as Colombia, Brazil, India, Canada and South Africa [3].

Based on the theory of change as a conceptual framework, the first steps for achieving behavioral change should include consumers’ attention and understanding of labels. Therefore, it requires investigating people’s knowledge and perceptions as well as their behaviors [4] or practices. Several qualitative studies have explored people’s understanding and perceptions of policies that include FoP labels. For instance in Australia, they found that the Health Star Rating was perceived as practical but were hesitant about the dissuasive impact of the scheme [5]. In Ecuador, the traffic light system could contribute to making informed decisions, but it may not lead to changes in consumption [6]. In Brazil, adult consumers were receptive to the introduction of FoP labels [7]. On the other hand, in Mexico, the GDA was mistrusted [8]. Most of this research has been conducted when the policy is being discussed or recently implemented. In Chile, we also conducted, after the initial implementation of the law, focus groups interviews with mothers of children from 2 to 14 yo (years old) [9] to have a deeper understanding of how the law was being perceived and diffused among families. We found that mothers understood and used the FoP warning labels, and that schools and daycares played a relevant role as law promoters. Subsequent studies have quantitatively shown that the initial implementation of the law was effective in reducing the amount of sugar and sodium in several packaged foods [10], in promoting a decline in purchases of “high in” foods, [11] as well as decreasing exposure to TV advertising of unhealthy foods [12].

The Chilean Labelling law was implemented in three phases in which the limits for defining unhealthy food products become increasingly strict [2]. It is hypothesized that as the limits become stricter with the subsequent phases of the law, the effects will become even larger. However, studies from other fields have also found that repeated exposure to health messages and warning labels may lead to message fatigue, inattention and reduced the effectiveness of health campaigns [13,14]. Thus, it is relevant to assess what the attitudes and perceptions of consumers are after the full implementation of the Chilean Labelling Law, which was in June 2019, 3 years after the initial implementation. It is important to note that full implementation of the law was achieved almost 8 months before the COVID pandemic arrived in Chile. Chile had one of the strongest responses to COVID, implementing long lockdowns periods to control the spread of the virus. Research has found that pandemic lockdowns altered people’s nutrition behavior in ways that vary depending on their socio-economic status and other characteristics [15], adding an extra layer of complexity to our analyses. Therefore, the purpose of this study is to qualitatively explore how the Chilean Labelling law has impacted people’s behavior and whether attention to and attitudes towards the FoP labels have evolved after four years of the implementation of the law. At the same time, because the pandemic and the lockdowns appeared when the Chilean food labeling law was fully implemented, we seek to investigate how the law and the pandemic interacted in shaping people’s nutrition practices.

## 2. Methods

We replicated the methodology and design of the study conducted in 2017 [9], when the first phase of the law had been recently implemented. In January 2021, when the three phases of the law had been fully implemented, and after the main lockdown of the COVID-19 pandemic had ended, we conducted nine focus groups of mothers of children aged 2 to 14 yo (N = 69). The mothers lived in 23 different districts of Santiago, Chile’s capital city. Therefore, they covered people from different backgrounds. Focus groups are a qualitative technique increasingly used in public health and policy evaluations [7,16] because they allow understanding of people’s environments, contexts, discourses, and practices [17] They also allow to observe how people interact, explore, and trigger each other’s arguments around a topic [18]. Finally, they provide insights into the underlying processes that explain why a policy might be working or not [19]. In this case, we wanted to explore mothers’ discourses, practices, and interactions with the law during the period of the pandemic.

Because of the COVID-19 restrictions, we conducted the focus groups online using the Zoom platform. The internet in Chile reaches over 82% of the population [20]. The focus groups followed a 3 × 3 stratification design based on socioeconomic status (higher, middle, and lower SES based on education, income, material goods) and age of at least one of their children (2–6 yo; 7–10 yo and 11–14 yo) given that the law is targeted to children < 14 y and because Chile is a highly stratified society in which nutrition practices are unequal and obesity rates are much higher among lower SES people [21]. In addition, dietary habits change according to the children’s age [22]. Therefore, we wanted to understand people’s experiences across different social classes and children’s development.

### 2.1. Participants

To obtain diverse backgrounds and experiences and make sure mothers were not related to each other, we hired a recruitment company. They recruited mothers from 23 out of the 35 districts in Santiago, Chile’s main city. We applied a screening questionnaire to exclude people who had worked in the marketing or food industry, select the participants, and assign them to different focus groups. The questionnaire included variables such as their age, children’s age, district, occupation, income, type of school their children attended (public, semi-private, private) and material goods (e.g., ownership of car, house, housekeeping service).

Although internet access is very high in Chile, we still wanted to make sure to recruit people from low SES, and not introduce a selection bias due to the online mode. Therefore, we paid special attention to the SES questions, and we carefully selected and assigned participants with diverse backgrounds to the different groups (see Table 1 for a description of the participants’ profiles).

### 2.2. Procedures

The questions asked in the focus group were created by a group of researchers who had participated in the previous study that come from different disciplines such as nutrition, epidemiology, public health, and communication. Based on our previous experience, other studies that evaluate the law and the extant literature on nutrition during the pandemic, we elaborated a set of 20 questions that explored their dietary habits before the pandemic, during the lockdown and after the lockdown, shopping practices and decisions, insights about their nutrition and dietary habits, and perceptions about the labels and other aspects of the law. For instance, a few questions were: What were your meal routines before the pandemic, during and after quarantines? Have you seen changes in the places and products you buy? What was the most relevant thing when shopping (e.g., healthiness, durability, price, availability, etc.? What are the sources of information you use to find out if something is healthy? Regarding the food law, do you pay attention to labels when shopping? Are there foods you used to think were healthy but you found out they are not? How so? Who influences the shopping decisions at home? These questions were a departing point for discussion. The saturation of themes was reached before completing the 9 focus groups.

The study received approval from Universidad Diego Portales’ IRB and the focus groups were led by one of the researchers and two research assistants who had participated in the 2017 study. Informed consent from participants was obtained at the beginning of the sessions. The identification information was anonymized. The focus groups sessions lasted 90 min approximately. They were transcribed by a professional transcriptions service and revised for accuracy by the researchers who attended the sessions.

### 2.3. Analytical Strategy

Following similar strategies conducted by several qualitative studies that relied on focus groups to evaluate nutrition policies that included FoP labels [5,6,7], and our own previous approach [9] the focus group transcripts were analyzed by two researchers (the leading and corresponding authors of this article) and a research assistant who had participated in the evaluation of the law since 2016. We followed a hybrid thematic analysis that integrated both deductive and inductive approaches [23] to develop coding categories that were based on previous literature and research questions as well as themes that emerged in the process of analysis. The codes were created after a reading process conducted independently by two researchers and then, also examined by the third investigator. Each paragraph was assigned at least one coding category. These coding categories were reorganized and collapsed by themes. In Excel spreadsheets, they were associated with quotes that best represented the themes. They were translated into English and revised by the team of researchers that co-author this study.

## 3. Results

***Label fatigue but acquired dietary knowledge:*** According to the mothers’ discourses, their attention to the labels decreased compared to when the law had been recently implemented, particularly among lower-SES mothers. They also complained about the oversaturation of labels.

For instance, Ignacia from a lower SES, asserts: “I feel that nowadays everything has a label, it is kind of hard to buy something with no labels”.

Elizabeth’s account also shows this fatigue: “I think we all got it at the beginning. Then, over time, it was hard for someone to keep looking at the labels. For us, the issue of labels is not an issue... they went to the background because everything has a label.”

However, at the same time, many people already know which products have more or fewer labels. They learned that several cereal bars, breakfast cereals or other products that were considered healthy before the law, are high in several critical nutrients.

Alejandra, from higher SES, said: “The brand NutraBien (Good Nutrition)... I like that brand… their muffins that are light and everything, they have labels. Some (breakfast) cereals that did not have labels, now they carry labels, the yoghurts did not carry labels and now only a few don’t. I like to drink this flavored Nestle coffee and it is high in sugar and has labels. The mayonnaise, we know it is high in saturated fats, but now it has two labels. So, one is like.. damn... same with buttermilks, margarines.”

***Nutrition consciousness and value of natural foods*:** Although the Chilean food law did not focus on the degree of food processing but, rather, on nutrients of concern [24], mothers’ discourses suggest that they equate FoP labels with more processed foods. At the same time, there is greater consciousness about less processed products and healthier nutrition.

For instance, Natalia from lower SES who has a child between 2 and 6 yo, explained that the saturation of labels led to valuing more natural nutrition: “I think in the beginning the issue of labels was a shock therapy. So, at the beginning we were all worried about the labels of the packages. Then, [we worried] about [the food] being more natural.”

Another mom of a preschooler shows that she started to value less processed foods when she realized that even foods that she considered healthy before the law had labels: “It happened to me when I used to buy these soda crackers. I said... ‘they are so ‘healthy,’ but they also had labels. Then, I was ‘Lord, what do we do?!’ So, I had to restructure. Now that I’m a mother, one of the main things I see is not to buy products that are so processed.”

Francisca, from middle SES, said: “Our diet has been very cautious, much more natural, we avoid processed things, everything that comes in a box or package.”

This becomes more evident during to the COVID-19 health crisis and lockdowns, in which many families started to cook. However, while the narratives of middle and higher SES mothers show that their cooking practices consider healthiness, in the discourses of lower SES mothers, healthiness did not emerge as a theme.

Valeria, high SES: “I prefer to go to La Vega [the Central Market] or the supermarket buy apples and make apple juice than going to the supermarket and buy a bottle of juice that is supposed to be natural but still has preservatives because to maintain a bottle in the gondola, it must have preservatives.”

Cynthia, from middle SES, and her family used to eat packaged cookies, but the pandemic changed this habit. “With the pandemic, and also for fear that [the cookies] had something, we started making them with oatmeal and nuts and that’s how the food routine began to change. I started preparing everything by myself. I made bread from scratch. Now we consume more fruits and veggies.”

Paula, from middle SES, also started cooking burgers at home but made of beans. “Our change in the pandemic that still continues, and yes, they like it, they don’t miss much buying junk food, are the chickpeas burgers… I became an expert in the kitchen trying to vary things.”

She started to cook using social media: “I started cooking healthier stuff, I started buying oatmeal or cooking with less sugar and more Stevia. I followed an Instagram [account] of healthy cooking, so most of the things became a bit healthier. If they wanted pizza, I cooked the dough, if they wanted fries, I’d bake them rather than fry them.”

Conversely, cooking practices of lower SES mothers during the pandemic concentrated on high-energy foods such as bread, pastries, and pizzas.

Patricia, from lower SES: “At dinner time, it’s always been piggy eating like hotdogs, pizzas, fries, but we don’t buy outside, we make here [home] almost all the junk food.”

Maria also explains that boredom led to overeating: “At least here in my house we have been eating more because of, as my eldest daughter says, boredom. From being bored one looks for something to entertain, making cookies, some dessert. It was more because of anxiety than other things.”

***Greater dietary knowledge collides with less access to healthy foods*:** Mothers’ greater understanding and acquired knowledge of the labels and better nutrition collides with the fact that healthier food is perceived as expensive and not available. This discourse was particularly relevant for lower SES mothers.

Laura, from lower SES, asserted: “[I first see] the price, not the labels. Besides, [foods with] labels are clearly cheaper, less healthy and healthier [food] is more expensive. And then you say, this one that is cheaper is hurting me, but this one is healthier and more expensive. So why don’t you [government] help us instead of replenishing with so many labels. For example, one kilo of apples is $1200 pesos [US$1.5] and I buy some applesauce at $300 pesos [35 cents] but it has a lot of labeling”.

In the same vein, Camila, also from lower SES, said: “I think that lately there is just more conscience, one knows that one should eat better, now that there are salads, fruits but still… it is hard to quit, to abandon habits. In other words, I’m eating less junk and things like that, but start eating like super healthy, it is still expensive and harder to find.”

In addition, the deprived economic context of the pandemic made these tensions even more evident. Elizabeth, **a** participant from a lower SES, explained: “Now in pandemic, the issue of labels was not a priority, and we prefer to look for the cheapest but of good quality. If it had three labels but was much cheaper than another with two labels, we started to prioritize buying cheaper to buy more.”

***School’s promoting role of the law was lost during the pandemic, particularly among low SES children.*** The focus groups that we conducted a year after the law implementation revealed that daycares and schools had been active promoters of children’s learning about the labels and changes in the quality of students’ snack eating. This had a spillover effect because younger children, particularly from lower SES, became agents of change in their households by requesting snacks with fewer or no labels as well as water rather than box juices or sodas. However, in Chile in 2020 and 2021 most public schools remained closed, which altered families’ nutrition habits and domestic routines [25].

Natalia, from lower SES, remembers that her children learned about labels in their school. “They had to bring stuff with few labels or no labels at all. So, one had to send them home-made things because they asked for things with no labels. They had a lot of knowledge about the labels, they know that fewer labels is healthier.” However, now that the schools are closed and “there were no snacks, I don’t even remember the labels”, Natalia added. Camila, also from lower SES, also talks about the role that schools had: “There was a time when the school gave us a talk about labels and nutrition because they didn’t allow to take anything with labels.”

Lower-SES children who go to public daycares or schools receive most of their meals at their facilities. Therefore, the schools’ closures meant a significant change in their dietary routines. Elizabeth explained: “We didn’t worry much about Anto’s food because she went to daycare. So, last year (pre-pandemic) she went all day so they gave her dinner and lunch. She came home and we have never had dinner, so she would come home and only drink milk.”

Denisse, from middle SES, complains about having her daughters at home and missing school because it gave them routines: “I think they were better when they were at the school, now they are too disorganized. They open the refrigerator several times a day. Being home make them eat.”

Finally, Camila, from lower SES, explained that in the beginning, when the law was first implemented, they tried to incorporate healthier snacks but it did not work out. Then, the attention to labels decreased and, after the schools’ closures, they do not feel forced to change the dietary habits anymore. “The problem is that we buy something and then we look at the labels and comes the remorse. But the truth is that we don’t pay attention too much because it didn’t work out to give healthier stuff, with no sugar. And now because she is at home and we don’t have to send food to school, I left them [the labels] aside.”

**Comparison of themes that emerged in the focus groups: 2017 vs. 2021.**

In Table 2, we present a comparison of themes that emerged in the focus groups conducted in 2017 vs. 2021. The comparison showed that in both years FoP labels were successful in providing information about food healthiness. Furthermore, FoP labels uncovered products that mothers considered healthy. In 2017 mothers paid greater—although with different gradation—attention to labels when buying, although they warned about possible saturation (see Table 2). In 2021, the discourses about paying less attention due to saturation and omnipresence were more prevalent.

Although in 2017, schools were perceived as promoters of change, in 2021 they were irrelevant in the mothers’ discourses because they were mostly closed in 2020. While marketing strategies were considered confusing or were unnoticed in the first study, they did not emerge as a relevant theme four years after the first implementation. Finally, mothers’ nutrition consciousness as well as lack of access to healthy food were only prevalent in the focus groups conducted in 2021.

## 4. Discussion

In this study, we replicate a qualitative investigation conducted after the implementation of the first phase of the Chilean food law to better understand how people’s knowledge, perceptions and practices about the law had evolved over time and how the regulation interacted with the perceptions and practices about nutrition and dietary behaviors during the COVID pandemic.

Several studies that relied on focus groups conducted during the discussion or right after the implementation of policies that included FoP labels have found that people showed a greater understanding of food healthiness [5,7,9]. However, this investigation showed that after four years of being exposed to FoPs, mothers had internalized the knowledge about labels and many of them knew which products carry more or fewer labels, but they experienced saturation and message fatigue. It is important to pay attention to this phenomenon. Despite the prevalence of experiencing message fatigue in people’s everyday lives, the communication and persuasion literature is recently –but increasingly-- concerned with the public’s fatigue towards health messages [26] including obesity-related messages [13]. It has been documented that repeated messages and overexposure might have a negative effect on effective health communication and recall [27]. It can also cause desensitization and avoidance [26]. This is problematic because exposure is a necessary condition to have a health message effect; therefore, reducing the messages is not a viable alternative [28]. In addition, in the case of Chile, most children had not attended schools for the entire year; therefore, they were not exposed to a key agent in the diffusion of healthy eating messages. Therefore, following guidelines of the literature on message fatigue [26], more viable options would be to identify those groups who are more susceptible to getting fatigued or desensitized. Social media campaigns allow to identify specific groups and reach them with tailored messages. In addition, persuasion theories suggest that slight variations in the messages are necessary, particularly when we want to reach populations that are not highly involved or motivated with the messages [29]. We believe these results showing the evolution of people’s perceptions after several years of the implementation of the law are enlightening for countries that are discussing or implementing regulations that include FoP labels.

Other policies have focused on changing food school environments [3]. In our previous study, we had found that schools were key promoters of healthy eating, by teaching younger children about healthy behaviors and using the labels as shortcuts to regulate snacking. Children, in turn, become agents of change for their families, thus extending the impact of the regulation. Moreover, this phenomenon was particularly relevant among lower socioeconomic status groups adding an equity component to the policy. In this investigation, we found that mothers recognized the negative implications of schools’ closures during the pandemic because that diffusion factor was missing. Children were not exposed to the messages and stopped requesting snacks with fewer labels or teaching the family members about healthy behaviors. Several authors have warned about the implications of school closure during the pandemic [30,31,32]; we add to this literature showing the implications for nutritional policies. In the particular case of Chile, schools might be a good place for doing a relaunching campaign of the labeling law including the use of warning labels for choosing healthier foods but also reinforcing the role of other food environment characteristics such as food marketing or food availability.

Mothers’ discourses about the marketing strategies prohibited by the law did not emerge as relevant in our focus groups. This is not surprising because we had already observed similar findings at the initial implementation of the law, although marketing restrictions interacted with other components of the regulation in promoting positive results. It is well established that the effects of food marketing usually operate through routes that require low attention and thinking and therefore, people might not even notice them. Disentangling how marketing regulations impact dietary behaviors is a very interesting and active subject of research [33].

Another interesting finding of our study is that despite the law did not focus on the level of food processing some mothers associated the labels with processed foods and therefore, the regulation has triggered a greater value of more natural foods. Several studies, including a randomized clinical trial, have shown the negative nutritional and health implications of the consumption of ultra-processed foods [34,35,36,37] leading to the recommendation of implementing policies that can decrease their consumption among the population [38]. Similarly, there is agreement that the intake of more natural foods such as fruits, vegetables, legumes, etc. needs to be promoted to ensure healthier and more sustainable diets [39]. The Chilean Labelling Law mandates that consumers need to be informed of foods high in nutrients considered critical for the emergence of nutrition-related chronic diseases (i.e., sodium, sugars, saturated fats) or energy by adding a warning message. However, some food products do not have high concentrations of critical nutrients or energy but do have additives that might be of concern [40]. Particularly, there is active research to establish the safety of using high concentrations of non-caloric sweeteners on food products consumed by high-risk populations such as pregnant women, infants and children [41,42]. Thus, it is interesting that despite this potential loophole of the Chilean regulation that does not prohibit non-caloric sweeteners, mothers understand that natural foods are preferred to packaged foods even if packaged foods do not have labels.

Our study also shows that the increase in nutrition knowledge is associated with increased recognition of other environmental barriers that hinder healthy eating, particularly among low SES families. Increasing physical and economic access to healthy foods as well as decreasing access to unhealthy foods have been highlighted as key aspects of promoting healthier diets [43]. The Chilean Food Law is unique because it promotes a package of actions that includes marketing regulations of unhealthy foods, improving information, and provision of healthier foods in the school environment [2]. However, an ideal package of food environment actions should also consider fiscal measures. Adding a tax on unhealthy foods and subsidizing healthy foods are policies that have been suggested to complement the law and further increase its impact [44,45]. This could be even more pivotal in the current economic crises derived from the pandemic.

Quantitative evaluations of the initial implementation of the Chilean regulation have been published [10,11,12] but the purpose of using a qualitative technique such as focus groups was to explore how people’s knowledge and perceptions regarding the law have evolved over 4 y of intervention. The technique used—focus groups—allows us to have a deeper understanding of people’s, contexts, discourses and practices as well as observe how mothers interact and trigger each other’s arguments about obesity, nutrition and the food law during the pandemic. Although the results of this study are not representative, generalizable or replicable, they are relevant for a policy evaluation because they provide vivid descriptions of the underlying processes that explain the effectiveness of a policy or lack of thereof [19] and will be very relevant for explaining ongoing quantitative analyses of the long-term impact of this regulation. Social desirability regarding nutrition or the opinion of the majority might affect people’s spontaneous participation and their willingness to express their candid opinions and practices. To avoid this possibility, as moderators we made sure everybody participated and made follow-up questions to explain any inconsistency. We conducted these focus groups by Zoom given the pandemic context. This mode might influence the ideal dynamics of the focus groups because it does not allow a fluid interaction among participants. Finally, we interviewed only mothers. Future research could include men’s perspectives.

## 5. Conclusions

This study, conducted after the full implementation of the Chilean food law and during the pandemic, contributes to our knowledge of the consequences of a comprehensive nutrition regulation after several years of being effective. It shows people’s fatigue with FoP labels, which suggests the need to identify groups that are more likely to get desensitized, develop ad hoc strategies, and reinforce targeted messages. The study also shows the importance of maintaining the schools open to promoting healthier diets among children and the need of developing complementary action to ensure that people have access to healthy foods. We believe these results contribute not only to improving Chilean nutritional policies but also to enlightening the discussion and implementation of similar regulations elsewhere.

## Figures and Tables

**Table 1:** Profile of mothers.

	Higher	Middle	Lower
Education	Complete College or higher	Complete college	95% complete high school 5% incomplete high school
Monthly income	CLP 2,500,000–5,000,000 (USD 3000–6000)	CLP 1,100,000–2,500,000 (USD 1300–3000)	CLP 250,000-750,000 (USD 300–950)
Material goods	100% own car, own house and housekeeping service	75% own car, 50% own house, 20% housekeeping service	0% own house, 0% own car, 0% housekeeping service
Districts	Vitacura, Las Condes, Providencia, La Reina	Peñalolén, Ñuñoa, La Florida, Pudahuel, San Miguel, Maipú, Providencia, Santiago, La Cisterna	La Florida, Puente Alto, La Granja, Renca, El Bosque, Maipú, Quinta Normal, Puente Alto, Macul
Occupation/ Profession	Lawyer, architect, business, Public relation officer, midwife, designer, real-state curator	Prevention, technician, executive secretary, IT, homemaker, property broker, teacher, accountant.	33% homemaker, manicurist, janitor, saleswoman, machine operator.

**Table 2:** Comparison of themes that were present in the focus groups of mothers 2017 vs. 2021.

	2017	2021
Attention to labels		
Knowledge and understanding (more labels, less healthy)	X	X
Uncovering role of labels	X	X
Gradational attention to labels (from no attention to being a shorcut when buying) or went unnoto	X	
Warnings of potential negative effect (omnipresence)	X	
Fatigue/disensitization/saturation		X
Schools’ role		
Schools as agent of change	X	
Schools are irrelevant because of closure		X
Marketing strategies		
Skepticism/confusion	X	
Unnoticed	X	
Nutrition consciouness		
Value natural foods		X
Presence of labels equals more processed food		X
Pandemic triggered a value in homecooking		X
Lack of access to healthy food		
Healthy food is more pricey		X
Lack of availability because of the pandemic		X

## Data Availability

The datasets generated and/or analyzed during the current study can be available from the corresponding author on reasonable request.

## Abbreviations

FoP:	front-of-package warning label
SES:	Socio-economic status
yo:	Years old
